# Selection of Specific Nanobodies against Lupine Allergen Lup an 1 for Immunoassay Development

**DOI:** 10.3390/foods10102428

**Published:** 2021-10-13

**Authors:** Yaozhong Hu, Chuan Zhang, Feier Yang, Jing Lin, Yi Wang, Sihao Wu, Ying Sun, Bowei Zhang, Huan Lv, Xuemeng Ji, Yang Lu, Serge Muyldermans, Shuo Wang

**Affiliations:** 1 Tianjin Key Laboratory of Food Science and Health, School of Medicine, Nankai University, Tianjin 300071, China; yzhu@nankai.edu.cn (Y.H.); 2120191296@mail.nankai.edu.cn (F.Y.); 2120201263@mail.nankai.edu.cn (J.L.); 2120201268@mail.nankai.edu.cn (Y.W.); 1120210672@mail.nankai.edu.cn (S.W.); bwzhang@nankai.edu.cn (B.Z.); lvhuan@nankai.edu.cn (H.L.); jixuemeng@nankai.edu.cn (X.J.); 2 College of Food Science and Engineering, TEDA Campus, Tianjin University of Science & Technology, Tianjin 300457, China; lhxp726@mail.tust.edu.cn (C.Z.); yingsun196@gmail.com (Y.S.); luyang@tust.edu.cn (Y.L.); 3 Cellular and Molecular Immunology, Vrije Universiteit Brussel, 1050 Brussels, Belgium; serge.muyldermans@vub.be

**Keywords:** lupine, crude protein content, nanobody, Lup an 1, sandwich ELISA

## Abstract

The declaration of lupine supplements is mandatory to avoid lupine allergy for sensitive individuals. However, reliable detection methods against lupine allergen remain critical to prevent the unintended consumption of allergen contaminated food. In this study, we have immunized an alpaca with lupine protein extracts and retrieved nanobodies (Nbs). Nevertheless, the target antigen has been recognized as Lup an 1, which has been classified as β-conglutin, and confirmed to connect with lupine allergy. After selection of the best Nb-pair, a sandwich enzyme-linked immunosorbent assay (ELISA) has been developed providing a linear range of 0.036–4.4 μg/mL with detection limit of 1.15 ng/mL. This immunoassay was confirmed by detecting the samples with spiked allergen, and a recovery from 86.25% to 108.45% with coefficient of variation (CV) less than 4.0% has been determined. Generally, this study demonstrated the selection of Nbs against allergen with crude protein content to develop the immunoassay for lupine surveillance in foods.

## 1. Introduction

Lupine belongs to the Leguminosae family in taxonomy together with other legumes including peanut, soybean and pea [1]. White lupine (*Lupinus albus*) is the most widely applied species having a protein content accounting for more than one-third of its dry weight, and it regularly serves as the common substitute of milk and soybean to increase nutritional value and flavor in gluten-free foods. Especially after the popularity of vegan meat, lupine extracts have been increasingly utilized to increase the protein content and dietary fiber [2]. However, lupine related allergies are on the rise to attract the global concern since the reported cases of allergic reactions, either against lupine allergens directly or from cross-reactivities to other legumes, especially peanut [3,4,5]. The clinical symptoms can be triggered in a short period of time after intake of lupine allergens with severe or even fatal reactions in sensitive individuals. Furthermore, the response including asthma, allergic rhinitis, urticaria, nauseas or gastrointestinal pains, and anaphylaxis can be variable in intensity and severity [6]. The allergic reactions caused by lupine have not been accurately counted and no death cases have occurred, yet. However, the increased addition of lupine isolates into foods should dramatically raise our concerns about lupine related allergies. The storage proteins, including the allergen of Lup an 1, have been identified as the main allergen causing allergic reactions [7,8]. Like other food allergies, there are still no specific curative strategies in case of lupine related allergies. The preventive treatment for patients with lupine allergy consisting of the implementation of an elimination diet avoiding all foods with lupine presence, is possibly the most appropriate approach to avoid these allergic reactions [3]. Therefore, the surveillance for lupine allergens in foods and the availability of reliable detection methods seems essential and feasible. These analytical techniques should guarantee avoiding intake of lupine allergens and should ensure the compliance with food labeling regulations, the disclosure of adulterations, and consumer protection.

To date, various detecting methods have been applied for the surveillance of lupine allergens in the food matrix by targeting different biomarkers including DNA and proteins, which encompass oligomer-based assays and immunoassays such as the quantitative real-time PCR and enzyme-linked immunosorbent assay (ELISA respectively) [9,10,11,12]. Antibody based immunoassay have been widely applied for the detection of allergens, and their accuracy mainly depends on the preparation and quality of allergen specific monoclonal or polyclonal antibodies. However, the distribution of allergens from precursor to matured formats, as well as the epitope variation after food-processing has complicated the testing efficacy of the immunoassay [13,14]. Therefore, novel antibody candidates with the capacity to detect lupine allergens under different conditions with higher specificity and accuracy should be introduced and evaluated. Camelidae species possess a unique functional antibody format known as heavy chain-only antibody (HCAb) with an antigen binding fragment that is restricted to one single variable domain of the heavy chain, referred to as VHH or nanobody (Nb) [15]. A straightforward and successful method has been developed to select specifically the Nbs against allergens [16]. More importantly, the unique characteristics of Nbs facilitate the recognition of their target antigens with relatively high affinity and specificity, with the potential to identify the unique epitopes that are difficult to be targeted by conventional antibodies [17,18]. The excellent characteristics of Nbs including the solubility and the stability under experimental conditions (e.g., high temperature, high or low pH and the presence of denaturants) qualified Nbs as a highly appropriate candidate for immunoassays [19,20].

**Figure 1:**
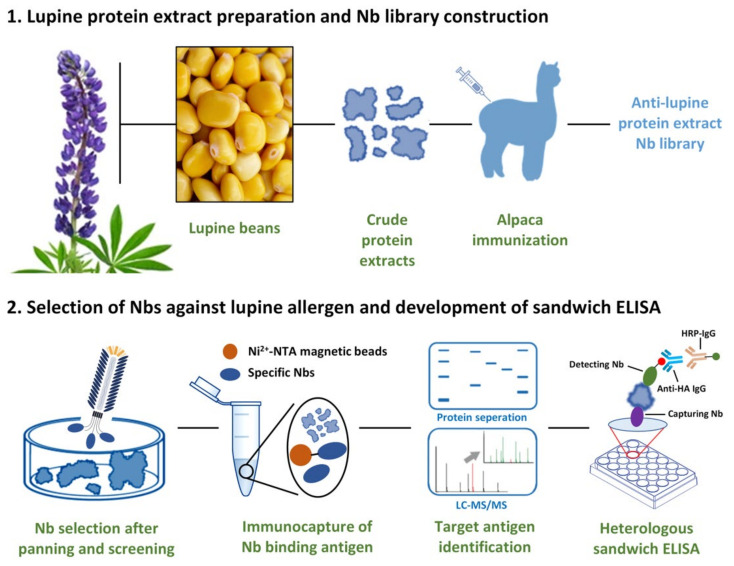
Illustration for the selection of specific Nbs with specificity against Lup an 1, and development of an immunoassay.

In this study, we proposed to employ Nbs as the detecting reagents in an immunoassay against lupin allergen (Figure 1). The target-specific Nbs were identified based on an unbiased strategy whereby crude lupine extracts were used instead of pure allergens. Immunization was performed by injecting an alpaca with general proteins isolated from lupines, and an immune Nb library was constructed to serve as the Nb repertoire to retrieve lupine-specific binders. The characteristics, including specificity, affinity and thermal-stability have been evaluated to verify the robust properties of the selected Nbs. The target antigen of the selected Nbs was determined by performing immunoprecipitation and mass spectrometry, and was subsequently identified as Lup an 1, which is classified as a lupine allergen protein. After selection of the Nb-pair giving the better response, a heterologous sandwich ELISA was developed with reliable applicability.

## 2. Materials and Methods

### 2.1. Crude Lupine Protein Extraction

The crude protein extracts from lupine were prepared based on the well-established protocol with minor modification [21,22]. Generally, gluten-free lupine powder was prepared with a grinder, and 30 g of lupine flour was de-fatted by supplementing hexane (500 mL) with agitating during 24 h at 4 °C with a magnetic agitator. After removing the hexane fraction, the remaining fraction was kept in a fume hood to eliminate the organic residue. The resuspension was then accomplished by adding 50 mmol/L Tris-HCl buffer (containing 1.0 mol/L NaCl, pH 8.0, 1:50 (*w*/*v*)), and incubate for 2 h with agitation. After centrifugation, the supernatant was then prepared as the crude extract of lupine. The solvent of crude extract was dialyzed against phosphate-buffered saline (PBS, pH 7.6). The collected liquid was concentrated by ultrafiltration and stored at −20 °C until further analysis. The concentration of the crude lupine extract was determined by using the BCA kit, and the protein distribution was analyzed by sodium dodecyl sulfate polyacrylamide gel electrophoresis (SDS-PAGE). 

### 2.2. Immunization and Construction of Nb Library

Immunization was performed by injecting the crude lupine isolates into a young alpaca (~2 years old) according to the well-established protocol with following modifications [23]. Generally, 0.4 mg of the crude extract was mixed with an equal volume of complete or incomplete Freund’s adjuvant, and then injected subcutaneously into an alpaca to elicit the immune response during a period of six weeks with one injection per week. Three days after the last boost, 50 mL of anti-coagulated blood was collected from the jugular vein, and placed under cool conditions for transportation. The alpaca was fed until the recovery, and the observation was proposed to guarantee the normal state. The peripheral blood monocytes were obtained with Lymphoprep™ Density Gradient Medium (STEMCELL, Canada) to facilitate the preparation of total RNA with TRIzol^TM^ Reagent (Invitrogen, Waltham, MA, USA) to serve as the template for synthesis of cDNA. PCR was organized to amplify the fragments with the encoding genes of VHH. The primers (CALL001: 5′-GTC CTG GCTGCT CTT CTA CAA GG-3′ and CALL002: 5′-GGT ACG TGC TGT TGA ACT GTT CC-3′) were used to produce the fragments containing the variable and constant domains (VH-CH1-CH2 fragment with the size of ~900 bp; VHH-CH2 fragment with the size of ~700 bp) in the first step of PCR. The amplified fragments were then separated after performing agarose gel electrophoresis with the fragments of ~700 bp excised and extracted by using the QIAquick Gel Extraction Kit (QIAGEN, Hilden, Germany) to serve as the template of the 2nd PCR. The primers (PMCF: 5′-CTA GTG CGG CCG CTG AGG AGA CGG TGA CCT GGG T-3′ and (A6E: 5′-GAT GTG CAG CTG CAG GAG TCT GGR GGA GG-3′) were used in this second round of PCR to amplify the VHH fragments containing Not I and Pst I sites to facilitate the insertion of the VHH amplicons into the pMECS phagemid vector after digestion with restriction enzymes of Not I and Pst I (NEB, Ipswich, MA, USA). The resulted recombinant phagemids were transformed into TG1 competent cells (Lucigen, Middleton, Wisconsin, USA) by electroporation. The diversity of the library was determined by plating a diluted aliquot of Nb repertoire transformed into TG1 cells on Luria-Bertani (LB) agar. For the construction of the library, the rest transformants were plated on large LB agar plates containing glucose and ampicillin. After overnight incubation, the colonies were scraped from the large plates and aliquoted as 1 mL/tube for stock in presence of 20% glycerol. The correct insertion of a VHH gene fragment was determined by colony PCR using primers GIII (5′-CCA CAG ACA GCC CTC ATA G-3′) and MP57 (5′-TTA TGC TTC CGG CTC GTA TG-3′) on randomly selected colonies. 

### 2.3. Bio-Panning

Enrichment of Nb displayed phages against lupine protein extract was obtained by consecutive rounds of bio-panning based on a well-established protocol [16]. In general, a representative fraction of immune library was inoculated into 2 × TY medium to grow until exponential phase. Then, 1 mL M13K07 helper phages (1.0 × 10^12^ *pfu*/mL) were added to infect TG1 cells, and to rescue the pMECS phagemid into virions containing the encoded Nb at the tip of the phage particles. After overnight incubation, the supernatant of the cultural medium containing the phage particles was collected after centrifugation, and phage particles were precipitated by incubating the supernatant with polyethylene glycol (PEG)/NaCl (1:10 (*v*/*v*)) on ice for 1 h. After centrifugation, the pelleted phages were resuspended with 1 mL of sterile PBS. The consecutive rounds of bio-panning were finalized using microtiter plates (Corning, Corning, NY, USA) immobilized with lupine proteins (100 μL, 50 μg/mL) for, and an ‘empty’ well (without lupine protein coat) served as ‘negative’ control. The wells were blocked with 200 μL of 3% skim milk (*w*/*v*), and then ~1 × 10^11^ *pfu* of phages with Nb-displayed were added into the wells with and without antigen coating, respectively, and incubated for 1 h at room temperature (RT). Unspecific phages were discarded after stringent washings steps with PBST (PBS containing 0.05% Tween-20, 10 times for 1st panning round, 20 times for 2nd and 3rd panning rounds). The remaining phages were collected after incubating the wells with TEA (100 μL, 100 mM triethylamine solution, pH 11.0), and then immediately neutralized in 100 μL Tris-HCl (1.0 M, pH 7.4). The enrichment for phages with antigen-specific Nbs was then determined by diluting 10 μL of the resulted phage with 90 μL of LB containing TG1 cells and plating on LB agar dishes. The remaining phage particles were allowed to infect fresh TG1 cells to amplify the phages used in the following panning round. 

### 2.4. Screening of Specific Nbs

To screen the Nbs positive in binding to the lupine protein extract, single colonies were randomly picked from the plates with enriched colonies after bio-panning and inoculated into 100 μL 2 × TY medium with supplementation of 100 μg/mL ampicillin, 2% (*w*/*v*) glucose, and 10% (*w*/*v*) glycerol in 96-well culturing plates (Corning, Corning, NY, USA). Then, 10 μL of cell culture were transferred to the corresponding wells of a 96-deep well plate (Axygen, Union City, CA, USA) containing 1 mL of 2 × TY medium after overnight incubation without shaking for incubating while shaking at 37 °C until the exponential growth phase reached. Isopropyl β-D-1-thiogalactopyranoside (IPTG) was added to reach a final concentration of 1 mM to induce the production of His- and Hemagglutinin (HA)-tagged Nbs after overnight incubation. Cells were incubated overnight and collected the next day by low speed centrifugation. The protein content including the induced Nbs were released after several freeze-thaw cycles, which were eventually applied to wells with or without coated lupine extract to identify the binding of antigen recognizing (i.e., positive) Nbs in a periplasmic enriched (PE)-ELISA. Wells coated with or without lupine isolates were designated as positive and negative in antigen binding, respectively. The signal corresponding to the binding properties of Nbs were detected at 405 nm after binding with mouse anti-HA IgG (1:3000) (Invitrogen, Waltham, MA, USA) and alkaline phosphatase (AP) conjugated goat anti-mouse IgGs (1:3000) (Invitrogen, Waltham, MA, USA). The colonies with the signal at least two-fold higher in the wells with antigen (positive) than the negative wells were considered as antigen-binders. These colonies that gave a positive signal were selected to prepare phagemid and to determine the nucleotide sequence of the Nbs.

### 2.5. Expression and Purification of Specific Nbs 

Selected Nbs were produced after transforming pMECS phagemids containing Nb genes into *E. coli* WK6 cells. The resulted WK6 cells were cultured in Terrific Broth (TB) medium supplemented with 0.1% (*w*/*v*) glucose, 100 μg/mL ampicillin and 2 mM MgCl_2_. Nb expression was induced by adding 1 mM IPTG into the cultural medium and after overnight incubation at 28 °C. Cells were collected after centrifugation, and then subjected to release the Nbs by osmotic shock. A two-step purification was applied to obtain the pure Nbs from the periplasmic extraction. Immobilized metal affinity chromatography (IMAC) was firstly organized to capture the Nbs by using HisPur^TM^ Ni-NTA Resin (Thermo Scientific, Waltham, MA, USA). After washing off the non-specific proteins, protein remaining on the resin was then eluted using 0.5 M imidazole in PBS. For the second purification step, the elution fractions were passed over on a size exclusion chromatography (SEC) on a HiLoad^TM^ 16/600 Superdex^TM^ 75 prepacked column (GE Healthcare, Chicago, Illinois, USA). SDS-PAGE was applied to determine the purity of the Nbs, and western blot was utilized to assess the presence of the His-tag and the identity with anti-His IgG (1:3000) (Invitrogen, Waltham, MA, USA) and horseradish peroxidase (HRP) conjugated goat anti-mouse IgGs (1:3000) (Invitrogen, Waltham, MA, USA). The concentration was determined by measuring the optical density at 280 nm (OD_280 nm_). The obtained Nbs were aliquoted and stocked at −80 °C with the concentration of 1 mg/mL until following evaluation. 

### 2.6. Identification of Target Antigen by Immuno-Capturing and LC-MS/MS

The target antigen of selected Nbs was identified by liquid chromatograph-mass spectrometer/mass spectrometer (LC-MS/MS) after immuno-capturing from the crude lupine extracts. In general, 5 μg of Nbs was added into 20 μL of HisPur^TM^ Ni-NTA Magnetic Beads (Thermo Scientific, Waltham, MA, USA), and incubated on a rotator for 1 h to form Nb-Ni-magnetic beads conjugates. The beads were collected with a magnetic stand and washed with PBST washing buffer (PBST with 50 mM imidazole; pH 8.0). Then, 300 μL lupine protein extract (1 mg/mL) was used to resuspend the conjugates, overnight at 4 °C with shaking to allow the immuno-capturing of target antigen by the Nb. After collection, the beads were washed with washing buffer, and the pelleted beads were then resuspended with Elution buffer (25 μL, PBS with 250 mM imidazole; pH 8.0) to elute Nb-antigen complex during 10 min incubation. The eluate was collected and SDS-PAGE was performed to separate the antigen captured by Nbs and stained with Coomassie-blue. Blank and negative controls were set up to confirm the band of the target antigen. The band in the gel slice was cut and subjected to prepare the peptides for analysis by LC-MS/MS (Waters Corporation, Milford, MA, USA). In general, the gel slice with target antigen was firstly de-stained completely from the Coomassie-blue. The gel was then digested to produce the peptides with trypsin, which will be analyzed by LC-MS/MS [24]. After analysis by LC-MS/MS, output data were aligned to identify the original sequences on the database of NCBI and Uniprot by using the peptide identification software of Proteome Discoverer 1.4 (Thermo Scientific, Waltham, MA, USA). Peptides were matched based on the control parameters of q-value and Posterior Error Probability provided with Percolator algorithm, and the false detection rate lower than 5% was guaranteed. 

### 2.7. Characterization of Nbs (Affinity, Specificity, Thermostability)

#### 2.7.1. Affinity

The affinity of selected Nbs was initially estimated by analyzing in ELISA the binding to their target antigens present within the crude protein extract. In brief, 100 μL of lupine extract (50 μg/mL) was immobilized in plates after overnight incubation at 4 °C. The remaining protein binding sites in the microtiter plates were blocked with 3% skim milk. Nbs were serially diluted in PBS and incubated with the coated proteins for 1 h at room temperature. The wells coated with PBS solution instead of Nbs serve as the blank to provide the background signal. The wells were incubated with the primary antibody of mouse anti-His tag IgG, and then the secondary antibody of HRP-conjugated goat anti-mouse IgG, respectively. 3, 3′, 5, 5′-tetramethylbenzidine (TMB) chromogenic solution was used to react with HRP, and 1.0 M H_2_SO_4_ was used to stop the color development. The optical density was determined at 450 nm with a microplate reader (Tecan, Männedorf, Switzerland). The concentration corresponding to half of the maximum signal defined the apparent affinity of the investigated Nbs.

#### 2.7.2. Specificity 

A western blot was performed to check the recognition of denatured antigens by the selected Nbs. Generally, the crude lupine protein extract was separated by SDS-PAGE, and then transferred to a nitrocellulose (NC) membrane (GE Healthcare, Chicago, Illinois, USA). The membrane was blocked with 3% skim milk, and cut into strips for incubation with a different Nb at a concentration of 1 μg/mL. After several washing steps with PBST, the wells were incubated with the primary antibody of mouse anti-His tag IgG, and then the secondary antibody of HRP-conjugated goat anti-mouse IgG, respectively. A blank without Nb was set up to confirm the absence of interaction between the antigen with primary and secondary antibodies. The color development was finalized after incubating with HRP staining solutions (A: 6 mL methanol with 18 mg Chloro-1-naphtol; B: 30 mL TPA solution (500 mL: 14.63 g NaCl, 1.4 g Trizma base, pH 7.5) with 19 µL H_2_O_2_). The antigen targeting of Nbs was visualized by the presence of the bands in western blot. 

#### 2.7.3. Thermostability

Thermostability of selected Nbs was evaluated with thermofluor using CFX Connect^TM^ Real-Time PCR System (Bio-Rad, Hercules, CA, USA). In general, Nbs were ultra-filtered to 2.5 mg/mL. A mixture system including Nb (15 μL) and SYPRO^®^ Orange Protein Gel Stain (5 μL, 1/100 diluted, Sigma-Aldrich, Burlington, MA, USA) was prepared to reach the final volume of 30 μL with PBS. The blank groups contained only PBS and SYPRO^®^ Orange Protein Gel Stain were set up for the base line control. Assessments were carried out in triplicate for each Nb. The program was installed to increase the temperature from 25 °C to 95 °C with increase of 0.5 °C/min, and the fluorescence was scanned for recording every 0.5 °C. The acquired data was analyzed with OriginPro 8 software to calculate the melting temperature (Tm) of focused Nb after non-linear fitting.

#### 2.7.4. Binding and Cross-Reaction of Nbs

Targeting capability and cross-reaction were determined by applying the selected Nbs in ELISA for binding the crude protein extracts of different origins (lupine, macadamia and peanut). Generally, the microtiter plates were coated with protein extracts (50 μg/mL) overnight at 4 °C. The plates were then blocked with 3% skim milk and washed with PBST. Then, Nbs at a concentration of 1 μg/mL were incubated for 1 h at RT. Wells incubated without Nbs were employed as the blank control. After rinsing with PBST to remove unbound Nbs, mouse anti-His IgG and HRP-conjugated goat anti-mouse IgG were used as the primary and secondary antibodies, and incubated for 1 h at RT. TMB substrate was employed to react with HRP. After termination with 1.0 M H_2_SO_4_ solution, the absorbance was recorded at 450 nm with a microplate reader. 

### 2.8. Nb-Mediated Heterologous Sandwich ELISA

#### 2.8.1. Expression and Purification of His-Tagged Nbs

For the development of a sandwich ELISA, His-tagged Nbs served as the capturing antibodies. These Nbs with only His-tag were prepared after periplasmic expression and purification steps. Firstly, the encoding genes of Nbs were subcloned into pHEN6c using restriction endonucleases Pst I and BstE II for periplasmic secretion after transformation into WK6 cells. Nbs were induced with 1 mM IPTG. After overnight incubation, cells were collected and subjected to osmotic shock for preparation of periplasmic proteins containing Nbs. The same purification steps (IMAC and SEC) were performed according to the steps mentioned above. The purity and the presence of His-tag were evaluated by performing SDS-PAGE and then western blot. 

#### 2.8.2. Selection of Nb-Pair and Optimization of Concentration

In this study, only His-tagged Nbs were employed as antigen-capturing antibodies, and Nbs with His- and HA-tag were used as antigen-detecting antibodies. The optimal Nb-pair was determined by checkerboard ELISA after comprehensive consideration about the response signals, the stability and cross-reactivity of the Nbs of interest. In brief, only His-tagged Nbs (10 μg/mL) were coated in microtiter plates. The wells were then incubated with 3% skim milk for blocking, and washed with PBST. Then, 100 μL of lupine protein extract (50 μg/mL) was added to the wells for capturing of the antigen by the pre-coated Nbs during incubation in 1 h. After washing steps, the detecting Nbs (10 μg/mL) were added to allow the interaction with the antigen protein captured by the capturing Nbs. Mouse anti-HA IgG and HRP-conjugated goat anti-mouse IgG were used as the antibodies primarily and secondarily. TMB was added for the reaction with HRP. After termination of the reaction with 1.0 M H_2_SO_4_, the optical density was recorded at 450 nm by a microplate reader. The optimal concentration of the capturing and detecting Nbs were determined by ELISA following the similar steps described above. Different concentrations of capturing and detecting Nbs (1, 1.25, 2.5, 5, 7.5 and 10 μg/mL) were utilized to quantify the antigen concentration. Nb-based sandwich ELISA was repeated at least twice to verify the consistency of the results. The calibration and linear standard curve were determined after non-linear or linear fitting. 

#### 2.8.3. Development of a Sandwich ELISA

Based on the best performing Nb-pair, and the optimal concentration for the sandwich ELISA, the standard curve was determined based on the absorbance value at 450 nm corresponding to the different concentration of lupine protein. Serial dilution of the lupine protein isolates was prepared and applied for the analysis by the sandwich ELISA. The obtained data could facilitate the determination of calibration curve after non-linear fitting, as well as the linear standard curve after linear fitting. The cut-off values including the limit of detection (LOD) and the limit of quantitation (LOQ) were determined by the data from blank wells with PBS only (*n* = 10), which defined the limit of detection (LOD) as the concentration of the mean optical density plus 3 times the standard deviations (SD), and plus 10 times the SD for the limit of quantitation (LOQ) [25]. 

Then, the specificity of the established method was evaluated against peanut, macadamia and lupine proteins for cross-reactivity analysis with the steps described above. The acquired data was analyzed to reflect the specificity of the method for the surveillance of lupine proteins.

#### 2.8.4. Detection of the Spiked Sample

The effectiveness of the developed immunoassay was determined against dairy products of skim milk. The spiked milk sample was prepared after supplementing different concentration of general lupine protein extracts. The samples spiked with antigens were cleared from the cream and precipitation after centrifugation. The obtained supernatant was diluted in 1000 times with PBS prior the detecting and applied for the antigen quantification by the established method to determine the recovery rate and coefficient of variation (CV), which provided evidence of the applicability of the developed immunoassay.

## 3. Results

### 3.1. Construction of an Immune Nb Library

Crude lupine protein extract was prepared with an acceptable efficiency. The molecular distribution of the proteins was visualized by Coomassie staining after SDS-PAGE (Figure S1), and their molecular mass ranged from 15 to 70 kDa, which fitted previous observations [26]. No significant variation was observed when proteins were separated under reducing or non-reducing conditions. The concentrated proteins with a size around 55 kDa could be observed and potentially reflected the distribution of conglutin, which has been identified as the main allergen source of lupinus.

An immune Nb library was constructed after immunizing a young alpaca with the crude lupine protein extract. The VHH repertoire was amplified by two rounds of nested PCR. The fragments of VH-CH1-CH2 or VHH-CH2 were identified based on their size of ~900 or 700 bp, respectively and the fragments of 700 bp were extracted and served as template for the 2nd PCR to amplify VHH gene fragments. After sub-cloning the VHH into pMECS vectors, the recombinant phagemids were transformed into *E. coli* TG1 to prepare the immune Nb library of about 1.44 × 10*^7^* colony forming units (CFU)/mL. The percentage of correct VHH insertion was determined by colony-PCR with randomly selected colonies to be 75% (data not shown). In summary, an adequate immune library against lupine protein extracts was constructed, which could be employed for retrieving lupine- specific Nbs.

### 3.2. Bio-Panning and Screening of Nbs

**Figure 2:**
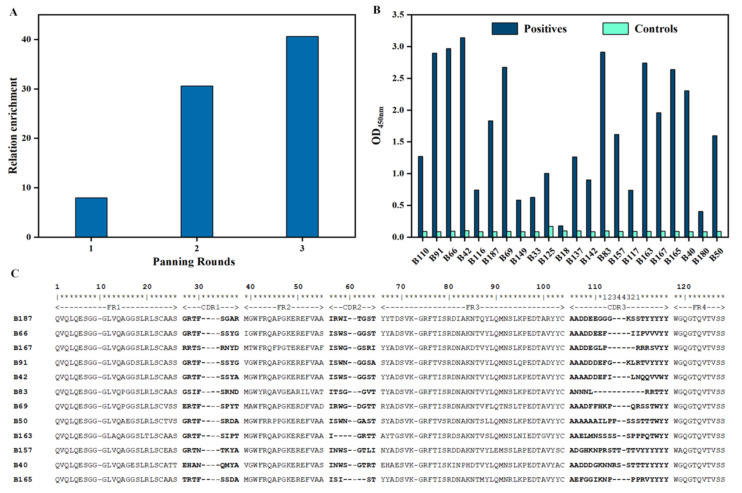
Selection and sequence of selected Nbs from the immune library. (**A**). Lupine protein-specific VHHs were enriched after each round of biopanning in comparison to control wells without antigen coated. (**B**). PE-ELISA for 190 colonies after three-rounds of panning. Only the colonies are shown that yielded an absorbance signal at least 2-fold higher than the signal of negative controls (no antigen coated in wells). (**C**). Twelve different amino acid sequences of lupine protein-specific VHHs were identified. The amino acid sequences of the nanobodies were aligned and their antigen-binding loops or complementarity determining regions (CDR) and framework regions (FR) are indicated.

Enrichment of Nbs recognizing lupine protein was accomplished by displaying Nbs at the tip of virus particles and followed by a round of bio-panning. The enrichment of each panning was evaluated by determining colony distribution from the phages collected from positive and negative, which demonstrated the strong increase of the enrichment from 8 to 40.6-fold (Figure 2A) reflecting an effective bio-panning for lupine protein specific Nbs.

To screen for Nbs with specificity for lupine protein, 190 single colonies (63 colonies from 1st round, 95 colonies from 2nd, and 32 from 3rd panning round) were picked randomly, cultured and treated to extract their periplasmic proteins, which contained Nbs possibly targeting the immobilized antigens in the following PE-ELISA. The results revealed that 22 colonies were exhibited with relatively a 2-time higher response in groups with immobilized antigen (positive wells) than without antigen (negative control) (Figure 2B). Further analysis of the amino acid sequence of the putative positive binders confirmed the presence of Nbs belonging to 12 different families. At least one representative family member (Nb B187, B66, B167, B91, B42, B83, B69, B50, B163, B157, B40, B165) (Figure 2C) was kept for further analysis. The uniqueness in the VDJ recombination gene has been indicated by predicting the corresponding regions with online server (VDJsolver) (Figure S2).

### 3.3. Purification and Specificity Analysis of Nbs

**Figure 3:**
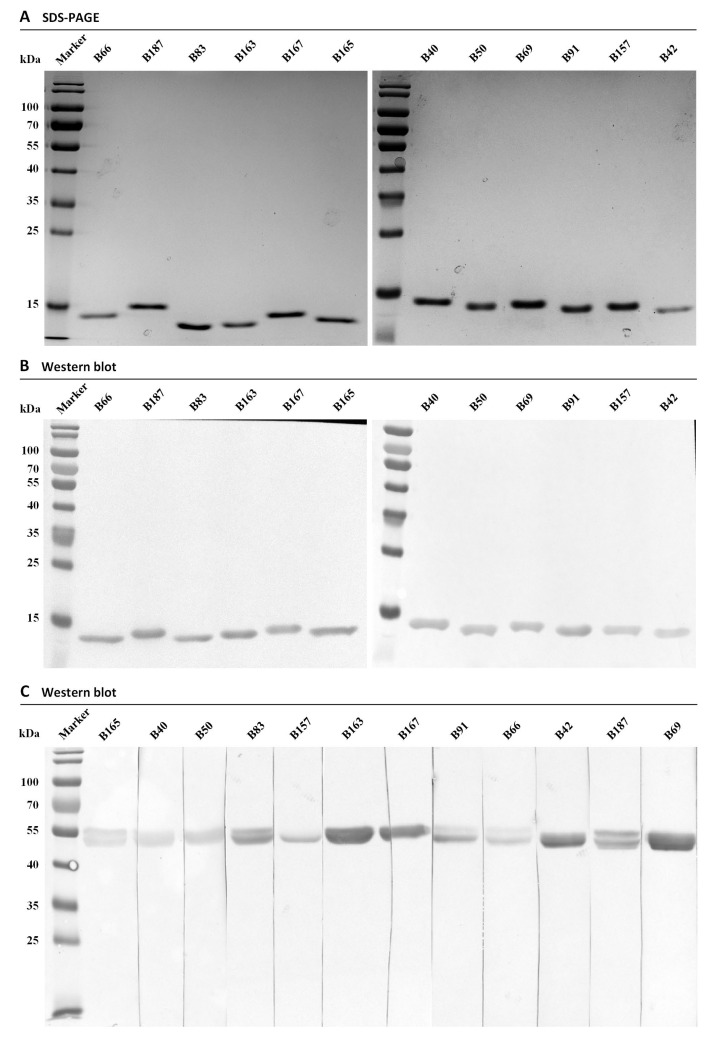
Confirmation and specificity analysis of selected Nbs. (**A**). The purity of Nbs with His- and HA-tag after expression and purification by IMAC and SEC was evaluated by SDS-PAGE. (**B**). The identify of His- and HA-tagged Nbs was evaluated by western blot after staining with mouse anti-His IgGs. (**C**). Antigen specificity of the Nbs was evaluated by western blot.

The recombinant plasmids of the PE-ELISA positive clones were extracted and transformed into E. coli WK6 cells. These cells were cultured individually and induced for periplasmic expression of their specific Nbs with IPTG. A two steps of purification including IMAC and SEC were performed to prepare Nbs with high purity. The yields of Nbs were confirmed to range from 5–20 mg/L culture medium. The purity of prepared Nbs was visualized by performing SDS-PAGE after Coomassie staining, and a single band of ~15 kDa, corresponding to the expected molecular mass of Nbs (Figure 3A) was detected. Moreover, the identity of Nbs was further confirmed by western blot revealed for the presence of His-tagged proteins (Figure 3B).

To confirm the targeting specificity of the Nbs selected for recognizing extracted lupine proteins, a western blot was performed to intentionally visualize the lupine protein bands after separation by SDS_PAGE under reducing condition. The results revealed that all the Nbs targeted an antigen(s) with a size around 55 kDa (Figure 3C), which was indicated either by a single band or by visualization of multiple bands. The results potentially demonstrated the recognition between the selected Nbs and a lupine protein, and confirmed the successful selection of specific Nbs.

### 3.4. Characteristics of Nbs

#### 3.4.1. Affinity of Nbs

The apparent affinity between the selected nanobodies and their target antigen was measured by saturated binding to immobilized lupine protein extracts in ELISA. The curves were illustrated after linear or non-linear fitting to determine the apparent affinity of Nbs (Figure S3). As shown in Table S1, the observed apparent affinities were recorded at 1, 8, 9, 14, 13, 16 and 24 nM for Nb83, Nb40, Nb91, Nb187, Nb167, Nb50 and Nb42, respectively, and a comparatively lower affinity was observed for Nb66, Nb163, Nb165, Nb69, and Nb157 with values of 180, 45, 31, 325 and 680 nM.

#### 3.4.2. Thermostability

The temperature resistance of Nb is of great importance to ensure the consistency during application. Herein, the thermal stability was assessed by thermofluor assay, and the curves were generated after non-linear fitting to illustrate the melting temperature (Tm), which reflected the thermal stability of the selected Nbs. As shown in Figure S4, the high thermal stability was observed for all Nbs, and the calculated Tm-values ranging from 52 °C to 77 °C were summarized in Table S1 (The Tm value of the Nb B40 was 60.45 ± 0.04 °C; The Tm value of the Nb B91 was 52.28 ± 0.05 °C). The obtained data demonstrated the conformity of the selected Nbs for the development of an immunoassay.

#### 3.4.3. Cross-Reaction of Selected Nbs

**Figure 4:**
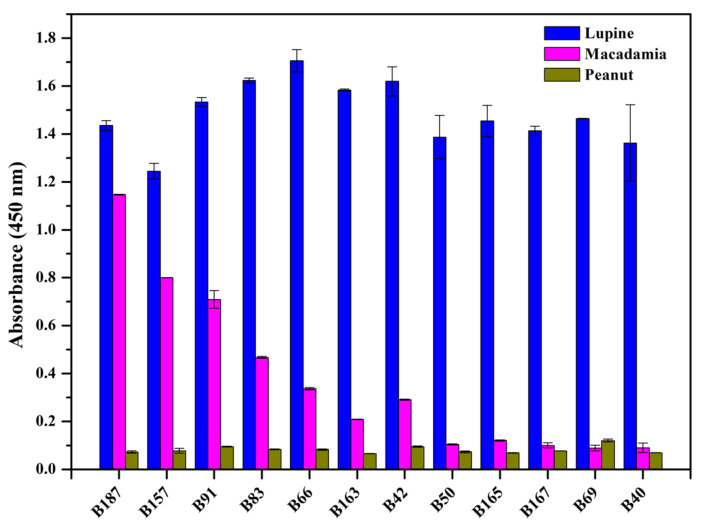
Cross-reactivity of selected Nbs. The cross-reaction with other antigens from macadamia and peanut has been determined by ELISA.

How the selected Nbs cross-react with other antigens was evaluated by determining the binding to the immobilized protein extracts from lupine, macadamia nut and peanut in an ELISA. A signal difference of 0.4 was accepted as threshold to make a distinction between significant and weak cross-reactivity, and a value difference of 0.2 was considered to differentiate between weak and no cross-reactivity. As shown in the Figure 4, four Nbs (B187, B157, B91, B83) were observed to significantly cross-react with macadamia protein extracts, and three Nbs (B66, B163, B42) had weak cross-reaction with macadamia protein. All the Nbs were demonstrated to possess any cross-reactivity with peanut protein extracts. The remaining Nbs (B50, B165, B167, B69, B40) were characterized to have a high specificity to lupine protein and no cross-reactivity was detected with macadamia or peanut protein extracts.

### 3.5. Identification of Target Antigen by LC-MS/MS

**Figure 5:**
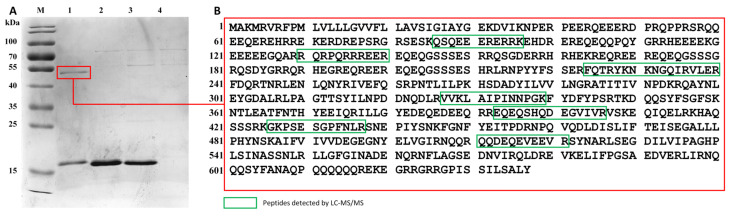
Identification of the target antigen of Nbs after immunocapture and LC-MS/MS. (**A**). The immunoprecipitate from crude lupine protein extract captured by Nb B187 via magnetic beads. The complex was separated by SDS-PAGE after unfold of di-sulfate bonds. Lane M: Pre-stained protein ladder; Lane 1: Nb B187 incubated with lupine protein extraction and Ni-NTA magnetic beads; Lane 2: Nb B187 incubated with Ni-NTA magnetic beads; Lane 3: an irrelevant Nb B147 (specific to the food-borne pathogen of Staphylococcus aureus (S. aureus)) incubated with lupine protein extraction and Ni-NTA magnetic beads; Lane 4: lupine protein extraction incubated with Ni-NTA magnetic beads. (**B**). Identification of the immunocaptured antigen by LC-MS/MS. The matched peptides from the cleaved bands (55 kDa) are indicated in the red rectangle. The target antigen was identified as Lup an 1 (UniProt sequence accession number: F5B8W3.1).

In order to identify the target antigen of selected Nbs, Nb B187 was employed to firstly immunocapture the antigen from the general protein solution, and then analyze the precipitated fraction by LC-MS/MS. Nb B187 was preferred due to its stability and high affinity. More importantly, multiple bands could be visualized after western blot with using Nb B187 as a probe, and the immunoprecipitation may relatively provide all the information of the target antigens after LC-MS/MS. Thus, Nb B187 was loaded on Ni-NTA magnetic beads to capture its cognate native protein antigen(s), for subsequent visualization after SDS-PAGE. The results facilitated the observation of the band of ~55 kDa after immunocapturing with B187 (Figure 5A, Lane 1). No significant immunocaptured protein was visualized in the control experiments, either in the test of B187 without lupine proteins (Figure 5A, Lane 2), or with an irrelevant Nb 147 (specific to the food-borne pathogen of Staphylococcus aureus (S. aureus)) with lupine proteins (Figure 5A, Lane 3) and blank without Nb or lupine proteins (Figure 5A, Lane 4). Bands of around 15 kDa were also present in samples containing the Nbs during immunocapturing. The antigen band of interest was excised, and then applied for the preparation of peptides after digestion with trypsin. The detected peptides by LC-MS/MS facilitated the match of the detected peptides with proteins in database, and the matches revealed a clear alignment with sequences from Lup an 1 (UniProt: F5B8W3.1, Figure 5B). Lup an 1 is classified as lupin β-conglutin form 7S seed storage globulin of vicilin-like proteins with a size distribution from 55 to 61 kDa. It has been identified as the allergen from lupinus that is recognized by IgE from lupin seed-allergic individuals [7]. Herein, the selected Nbs can be potentially applied for the detection of trace Lup an 1 in food matrix. 

### 3.6. Development of a Heterologous Sandwich ELISA

**Figure 6:**
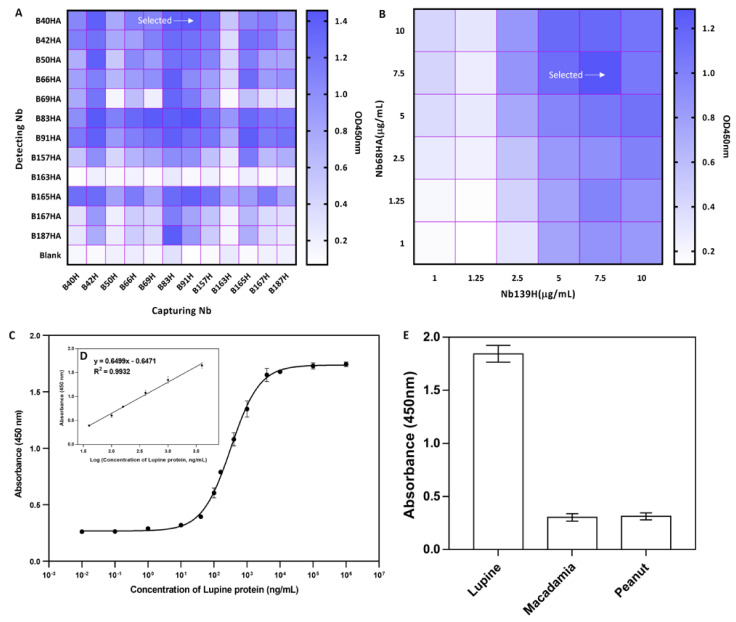
Development of a heterologous sandwich ELISA for detection of Lup an 1 allergen. (**A**). Selection of Nb-pair with best performance including a high response and low background. Nb B91H was selected as the capturing antibody, and Nb B40HA was selected as the detecting antibody. (**B**). The concentration of capturing and detecting Nbs was optimized based on the response and background. The concentration of 7.5 μg/mL was selected for both the capturing and detecting antibodies. (**C**). Calibration curve of the developed sandwich ELISA for detection of lupine allergen. (**D**). Linear standard curve of the established immunoassay. (**E**). Specificity and cross-reactivity of the developed sandwich ELISA. Data are indicated as mean ± SD (*n* = 3) with triplicates.

Being the main allergen of high abundancy in lupine, Lup an 1, it can possibly be employed as the detecting marker to indicate presence of trace amounts of lupine components in food. Thus, a heterologous sandwich ELISA was proposed for the surveillance of Lup an 1 in food samples. For the establishment of such immunoassay, analyte specific capturing and detecting Nbs (referred to as Nb-pair) that recognize (preferably) different epitopes on Lup an 1 are required. In this study, Nb with only His-tag was used to capture the antigen, and Nb with His- and HA-tag served as antigen-detecting antibody. Nbs with only a His-tag have been prepared after subcloning the Nb gene into pHEN6c expression vector that lacks the HA encoding gene fragment. The best performing Nb-pair was determined comprehensively based on the response in ELISA and the background value. As shown in Figure 6A, five different combination of Nbs (capturing/detecting Nb: B83H/B187HA, B69H/B83HA, B91H/B83HA, B42H/B83HA, and B91H/B40HA) were observed with relatively higher response. After comprehensive investigation, B91H/B40HA was selected as the pair used to establish the sandwich ELISA. 

The concentration of capturing and detecting Nbs was optimized to ensure the best possible performance in the sandwich ELISA. The concentration of Nbs has been determined based on the condition providing the highest signal and the lowest background. As shown in Figure 6B, the optimal concentration of 7.5 μg/mL for both capturing and detecting Nbs has been verified to produce the highest response signal and lowest background.

Based on the conditions decided in previous investigation, the surveillance parameters including LOD and LOQ of the developed immunoassay has been evaluated by detecting the Lup an 1 antigen. The titration curve was formulated against the crude lupine preparations at concentrations between 10*^−2^* and 10*^7^* ng/mL (Figure 6C). The standard curve has been determined with the fitted equation of y = 0.6499log(x)-0.6471 (R*^2^*= 0.9932; *n* = 3) (Figure 6D). The linear extension was calculated as the range of 0.036–4.4 μg/mL based on the Lup an 1 in general extract with 1.15 ng/mL for LOD and 29.75 ng/mL for LOQ.

### 3.7. Cross-Reactivity of the Developed Sandwich ELISA

Since Nb B91 exhibited weak cross-reactivity with macadamia protein extracts, we had to evaluate the cross-reactivity for this antigen with the established sandwich ELISA to verify its applicability. The macadamia or peanut proteins were detected by this sandwich ELISA, and the results revealed the absence of any significant cross-reactivity (Figure 6E). The results demonstrated the detecting efficiency and applicability of this ELISA with specificity for Lup an 1 lupine allergen.

### 3.8. Analysis of Spiked Sample

The applicability of the established immunoassay in food sample was assessed by supplementing different concentration of lupine protein extracts in dairy. The samples were then centrifuged to collect the supernatant, which was diluted 1000 times in PBS before testing to reach the final spiked concentration of 0.04, 0.4, and 4 μg/mL. As summarized in Table 1, the recovery of the lupine allergen protein in skim milk was determined from 86.25% to 108.45% with CV less than 4.0%, which demonstrated the reliability of the established immunoassay to detect lupine allergen in food matrix.

## 4. Discussion

In this study we prepared a crude lupine protein extract to immunize an alpaca and to select specific Nbs against lupine antigens. The strategy is quite straightforward while avoiding efforts for lupine protein purification, it is applicable for the study of unspecified targets for which prior knowledge is lacking. Various studies have utilized this strategy to retrieve specific Nbs against proteins of different origins, such as the selection of Nbs against the proteins from trypanosome secretome or Arabidopsis seed proteins [27,28]. The successful selection of specific Nbs and the identification of target antigen would be important to ensure research progression and further applications of Nbs. 

In this study, an immune Nb library has been constructed and 12 Nbs binding to proteins from the lupine protein extract have been retrieved after panning and screening of individual clones. In the future, this immune library could also be employed for the selection against purified lupine proteins to obtain novel binders against specific lupine targets of interest [27]. However, when retrieving binders after panning on crude protein extracts, it is challenging to confirm the Nb targeted antigen. Here, we solved this difficulty to identity the antigen of the obtained Nbs by first immunocapturing the natural antigen from the crude protein mixture via Nb conjugated Ni^2+^-NTA magnetic beads. In the next step, the immunocaptured antigen was analyzed by LC-MS/MS. Subsequently, a western blot could confirm the identity of the antigen with the selected Nbs (Figure 3C). In case of Nb B187, a few bands were revealed after western blot using this Nb as a probe, while only one protein was immunocaptured on Nb loaded magnetic beads, which was identified as Lup an 1, a β-conglutin (accounting for 43–45% of lupine protein content) from globulins (approximately 87% of the total protein content) in lupinus, and always connected with unfavored allergic diseases [6,29,30]. Hence, we speculated that the additional bands after western blot could originate from decorated Lup an 1 with slightly different mobility in SDS-PAGE, or from cross-reaction with other lupine fractions. Nevertheless, this Nb should have the potential to monitor trace levels of lupine components in a food matrix through detection of Lup an 1. In summary, it has been demonstrated that immunization and selection on crude protein extracts can yield high affinity Nbs with specificity against a lupine allergen.

To date, numerous detection techniques against lupine allergen have been developed including the DNA-based methods, and protein-based immunoassays [31]. Demmel et al. synthesized primers of internal transcribed spacer 1 (ITS1) gene from *L. angustifolius* to develop a DNA-based assay with LOD of 0.1 mg/kg (lupine flour/foods) [9]. Scarafoni et al. reported a real-time PCR based detection strategy against *L. albus* CγA32 gene (Lup a γ-conglutin), and possess the detection limit lower to 7 pg of lupine DNA [32]. However, DNA-based methods are considered as an indirect approach since the target protein is not detected. The residual level of Lup an 1 is not reflected after DNA analysis. In general, an immunoassay to detect antigen directly, can provide the direct information of the trace lupine allergen contamination in food sample. It can be more sensitive, and accurate in comparison with DNA-based methods. Different types of ELISA including indirect, direct and sandwich ELISA have been generated using polyclonal anti-lupine IgG or IgY antibodies, to facilitate the surveillance. Holden et al. developed a sandwich ELISA with polyclonal rabbit anti-lupine antibodies reaching a detection limit of 1.0 mg antigen/kg [33]. In another immunoassay with polyclonal and monoclonal antibodies, severe inconsistencies were observed after checking 112 food samples declared to contain (or not to contain) lupine [12]. In addition, the polyclonal antibodies or antisera reagents could also cross-react with unintended antigens. Therefore, in this study we developed a heterologous sandwich ELISA with Lup an 1-specific monomeric Nbs. The Nb-based immunoassay has been demonstrated to indicate the allergen with good reproducibility and sensitivity in detecting antigen in spiked samples. Whereas, the cut-off values of the developed immunoassay were determined by immobilizing the general protein content, further evaluation could be performed to provide more accurate parameters by spiking milk with purified Lup an 1. In summary, this study obtained specific Nbs against food allergens for development of a specific and sensitive immunoassay.

In conclusion, the present work successfully demonstrated the preparation of specific Nbs targeting to allergen protein by using crude protein extracts. Nbs categorized in 12 families were retrieved to recognize presumably the same antigen, which also verified the dominant response to the target. The antigen was then determined as Lup an 1, which has been classified as the allergy from lupinus and contributes to the allergic reaction in individuals. A sandwich ELISA has been established to provide the surveillance of lupine allergen Lup an 1 in food. More importantly, it is also imperative to conjugate these Nbs with other detecting techniques for the development of strategies with high applicability, such as sensor-based immunoassay. Further studies can be organized to identify the epitopes, as well as the structural information of Lup an 1.

## Figures and Tables

**Table 1:** Recoveries of lupine allergen in milk samples tested by sandwich ELISA.

Food Sample	Spiked Concentration	Detected Concentration	Recovery ^1^	CV ^2^
	μg/mL	μg/mL	%, *n* = 3	%
**Skim Milk**	0	ND ^3^	— ^4^	—
	0.04	0.04 ± 0.0148	108.45	2.42
	0.4	0.38 ± 0.0151	97.25	1.23
	4	3.45 ± 0.0592	86.25	3.24

^1^ Each assay was repeated three times. ^2^ CV: ratio of the standard deviation. ^3^ Non-detected. ^4^ No data provided.

## Data Availability

Not applicable.

## Supplementary Materials

The following are available online at https://www.mdpi.com/article/10.3390/foods10102428/s1, Figure S1: The crude lupine protein was extracted and analyzed by SDS-PAGE, Figure S2. Alignment of the VDJ recombination gene of Nbs, Figure S3: Apparent binding affinity of selected Nbs, Figure S4: Thermal stability of selected Nbs, Table S1: Properties of selected Nbs.
