# Supplementary material for: Selection of Specific Nanobodies against Lupine Allergen Lup an 1 for Immunoassay Development
Source: Foods. 2021 Oct 13;10(10):2428. doi: 10.3390/foods10102428 (PMC8536012; doi:10.3390/foods10102428)
Supplement: Supplementary file 1 [file foods-10-02428-s001.zip › foods-1405333-supplementary.pdf]

Selection of specific nanobodies against lupine allergen Lup an 1 for immunoassay development

Yaozhong Hu[1, #], Chuan Zhang[2, #], Feier Yang[1], Jing Lin[1], Yi Wang[1], Sihao Wu[1], Ying Sun[2], Bowei Zhang[1], Huan Lv[1], Xuemeng Ji[1], Yang Lu[2], Serge Muyldermans[3], Shuo Wang[1, *]

1. Tianjin Key Laboratory of Food Science and Health, School of Medicine, Nankai University, Tianjin 300071, China.

2. College of Food Science and Engineering, Tianjin University of Science & Technology, Tianjin 300457, China.

3. Cellular and Molecular Immunology, Vrije Universiteit Brussel, 1050 Brussels, Belgium.

[#] These authors (Y. Hu and C. Zhang) contribute equally.

[*] Corresponding author,

Email: wangshuo@nankai.edu.cn

Tel.: +86 22 85358445

Table S1. Properties of selected Nbs

**Nbs**	**MW**	**pI**	**Tm value** [a]	**Affinity** [b]
	kDa	—	℃	nM
**B40**	14.10	5.88	60.7 ± 0.06	9.6 ± 2.31
**B42**	13.50	6.39	69.7 ± 0.03	27.0 ± 5.20
**B50**	13.58	9.07	53.4 ± 0.07	20.2 ± 3.37
**B66**	13.34	5.31	77.9 ± 0.07	170.5 ± 21.9
**B69**	13.88	8.66	59.7 ± 0.18	404.7 ± 20.9
**B83**	12.52	9.34	69.1 ± 0.04	1.4 ± 0.81
**B91**	13.66	4.98	67.4 ± 1.00	17.2 ± 6.95
**B157**	14.16	8.57	66.3 ± 0.11	743.4 ± 96.9
**B163**	13.09	8.70	63.4 ± 0.04	7.5 ± 3.11
**B165**	13.40	9.17	62.3 ± 0.09	44.0 ± 2.34
**B167**	13.74	9.01	58.6 ± 0.02	14.5 ± 2.48
**B187**	13.80	8.91	72.5 ± 0.05	23.0 ± 6.36

[a]: Thermal stability of selected Nbs were represented as Tm value generated from 3 repeated assay, with triplicates for every assay, and the data was illustrated as mean ± SD (n = 9).

[b]: Apparent affinity of selected Nbs were represented as mean ± SD, and the test was repeated for 3 times (n = 3).

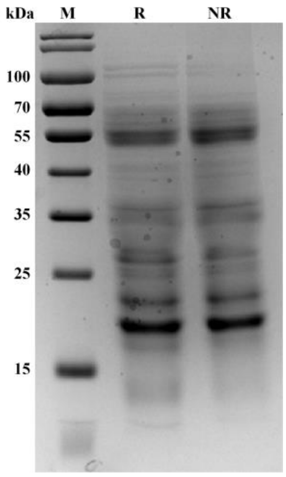

Figure S1. The crude lupine protein was extracted and analyzed by SDS-PAGE. SDS-PAGE was performed under the condition of reducing (lane R) and non-reducing (lane NR) to indicate the size and distribution of the general lupine protein. The labeled standard molecular weight (kDa) of the protein is on the left side (lane M).

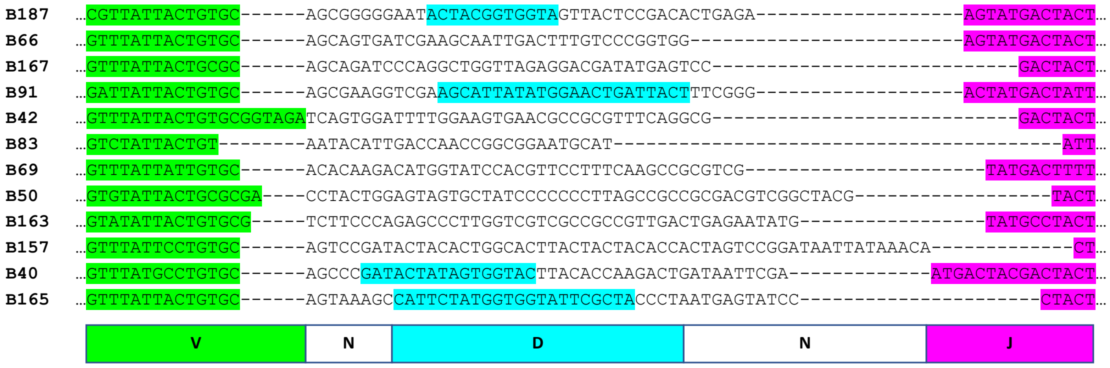

Figure S2. Alignment of the VDJ recombination gene of Nbs.

The VDJ segments have been predicted with the online software of VDJsolver (http://www.cbs.dtu.dk/services/VDJsolver/). V represents the V segment, N as the addition at the N-terminus, D as the D segment, and J represents J segment.

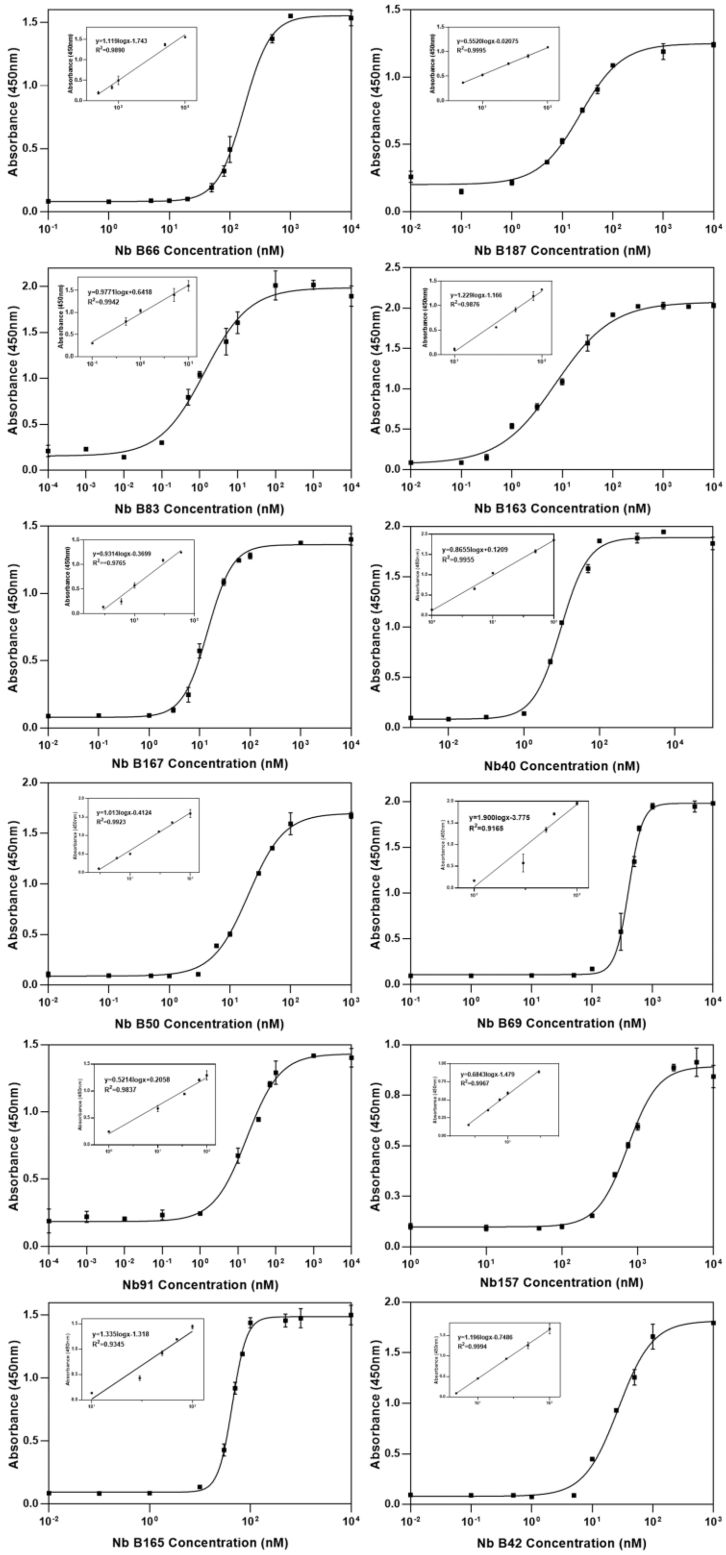

Figure S3. Apparent binding affinity of selected Nbs.

All data plotted are expressed as mean ± SD (n = 3). Repeated at least 3 times for every test.

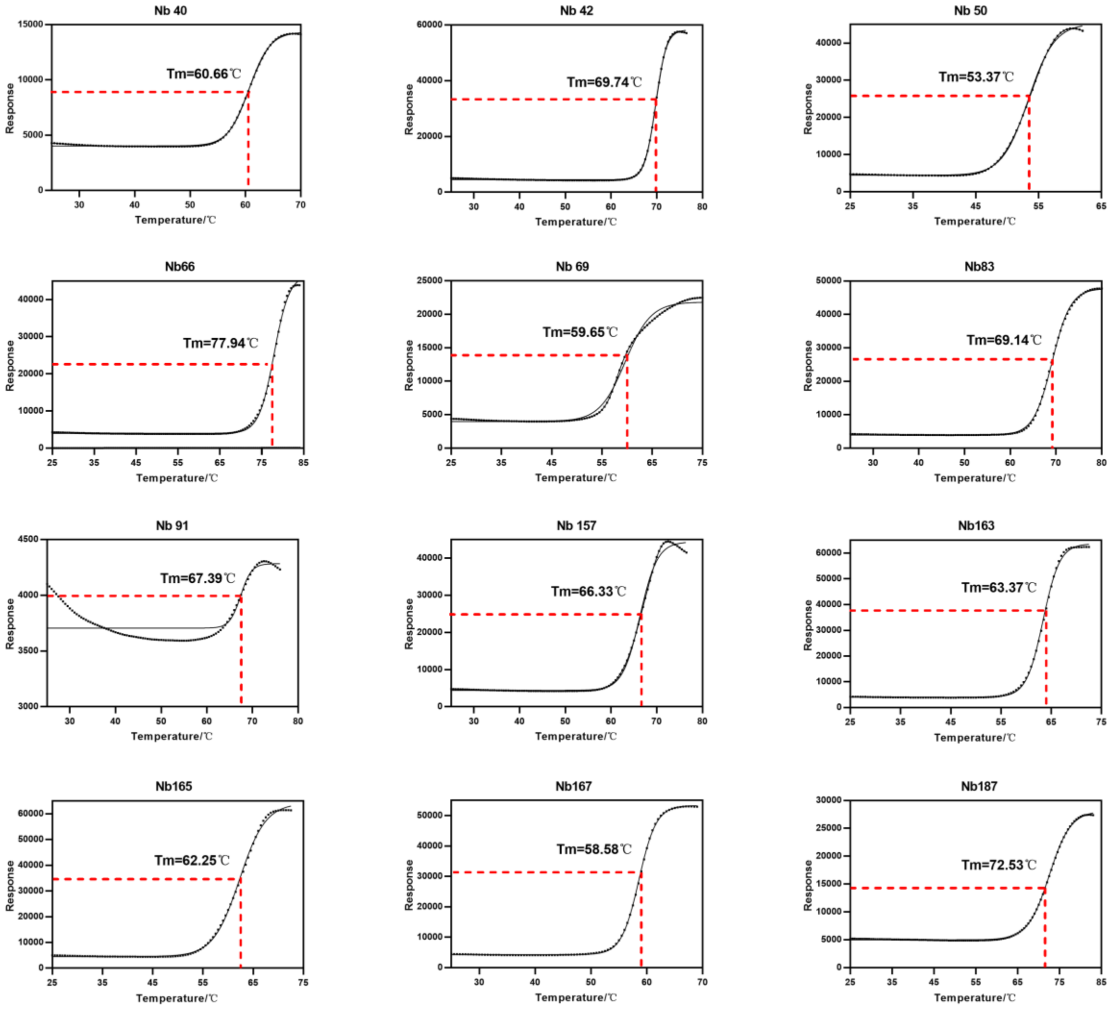

Figure S4. Thermal stability of selected Nbs.

All data plotted are expressed as mean ± SD (n = 3). Repeated at least 3 times for every test.
